# America and Its Charities: A Suggestive Experience

**Published:** 1887-01-29

**Authors:** 


### America and its Charities: A Suggestive Experience.

Many of our English readers have possibly never heard of the useful Society which, under the title of "The Associated Charities of Boston," has for eight years been doing much good work in that great American city. We may, therefore, briefly state that its object is to secure co-operation amongst the different Boston charities, with a view to raise the indigent above the need of relief, prevent begging and imposition, diminish pauperism, and encourage thrift, self-dependence, and industry. To accomplish these objects it has established sub-committees, or Conferences, in twenty-five wards of the city, the members of which thoroughly investigate the case of every applicant for relief, and deal with it in what appears the wisest and best manner. Thus, for some employment is obtained, and to others money gifts or small presents for the decoration of their homes, and their own consequent encouragement, are given, all relief being conditional upon good conduct and progress. We have just received the seventh annual report of the Society, which seems to be admirably compiled, and contains much that is interesting. The following passage refers to the abominable practice of sending children to beg in the streets, to which we alluded in our leading article of the 15th January, and we quote it as a strong confirmation of our own views then expressed:—"One of the saddest sights in a great city is to see the children, both boys and girls, who either beg outright or conceal a more successful begging under the pretence of selling some trifling article—matches, sleeve-buttons, or the like—often late at night, and sometimes in winter, wretchedly clad. The first impulse of every kind heart is to buy of them or give them money. But, alas! this only insures their being kept in this cruel life of present suffering, too often leading to a most wretched future career. Many of these children come from homes where parents could support them if they chose, but have learned this shameful art of sending them out to prey on the public sympathies by their sufferings."

**Objects and Report of the Society.**

**The Labour Test for Women.**

The Society has established a woodyard as a labour test for male applicants, and finds it to produce such admirable results, that some similar plan for women is strongly recommended in the report. "The supreme reform of out-door relief is to dispel the tempting impression that aid is easy to get; that, if you tell a piteous story, and show an empty cupboard or coalbin, you can get coal and food given gratuitously. When men found that, applying for aid, they were sent to saw wood, the number of applicants fell off, preferring to select their own—no doubt a better—field of labour. When women are met with a like offer of work, a great reform will be effected."

**A Dishonest Landlord.**

That the Society does not shrink from extreme measures in defence of its deserving *protégés*, is evidenced by the following narrative:—"Late in the fall of 1885 a family was referred to us, consisting of a man and wife and four children, the eldest a boy of fourteen. Relatives had aided as long as able. A situation was obtained for the boy with an architect at three dollars a week, and sewing for the woman from the Ladies' Union Charitable Society. Our agent learned that a former landlord had retained the woman's sewing-machine for rent, although the woman had receipts showing that all the rent due had been paid. The case was given to our honorary counsel, who immediately wrote the landlord a letter requesting an interview. His wife responded, and said the machine was abandoned by the family upon their removal; and she declined to restore it until storage had been paid. A suit was begun to recover the value of the machine, and, upon trial in the municipal court, judgment for fifty dollars and costs was rendered for the plaintiff. An appeal was made, but never prosecuted; and upon the landlord's refusing to pay the amount of the execution, his real estate was levied upon, and advertised to be sold, whereupon the defendant came forward and settled the bill in full. The woman purchased a new machine, sewing was obtained for her, and she is now earning good wages by dressmaking. The husband, who up to this time had been lazy and inefficient, aroused himself, opened a small fish-market, and has now several good paying customers. The boy is still at work, and his employer, who is now in Europe, has promised to bring him a set of architect's instruments for a present. The family are of course self-supporting."

**The Effect of Poor-law Relief.**

In another part of the report, however, we find a story of a very different kind from the foregoing:—"We have strong proof of the demoralising effect of relief in the example of a family who have been accustomed to receive city help for a number of years. Our visitor was surprised, in calling one day, to find them in possession of a piano. On making inquiries about it, she was told that the children had saved small sums of money, given them from time to time, until they had sufficient to purchase this piano, which, although not an expensive one, yet cost a large sum for them. The family were quite innocent in the matter, not seeming to feel that they had done any wrong in making such a purchase while receiving aid from the city."

**Finance and Method of the Society.**

The receipts of the Associated Charities for 1885-86 amounted to $17,554, and the expenditure to $17,241, thus leaving a balance of $313 to be carried forward, whilst the funded property consists of a sum of $2,400, details of which are duly given in the report. In conclusion, let us extract one more quotation, which gives a sound and practical view of the method to be adopted by Societies of this nature:—"We have endeavoured to lay great stress upon thorough investigation of cases. The possibility of imposture is not so much to be guarded against as the constant danger of mistakes, arising from a misunderstanding or an incomplete understanding of the character and needs of the families. Two-thirds of the errors in charity work arise from misinformation or lack of information. In thorough preliminary investigation, followed by an intelligent, sympathetic searching into the facts on the visitor's part, lies our chief strength as a practical body of scientific workers. As well ask a physician to prescribe for a stranger without seeing him, or a lawyer to argue a case without reading the testimony, or a farmer to plant his corn in an unploughed field, as to endeavour rightly to aid a poor family without full knowledge of its antecedents."

